# Changes in the sublingual microcirculation following aortic surgery under balanced or total intravenous anaesthesia: a prospective observational study

**DOI:** 10.1186/s12871-018-0673-7

**Published:** 2019-01-05

**Authors:** Silvia Loggi, Nicoletta Mininno, Elisa Damiani, Benedetto Marini, Erica Adrario, Claudia Scorcella, Roberta Domizi, Andrea Carsetti, Simona Pantanetti, Gabriele Pagliariccio, Luciano Carbonari, Abele Donati

**Affiliations:** 1 Anaesthesia and Intensive Care Unit, Department of Biomedical Sciences and Public Health, Università Politecnica delle Marche, via Tronto 10/a, 60126 Torrette di Ancona, Italy; 2 Unit of Vascular Surgery, Azienda Ospedaliera Universitaria “Ospedali Riuniti Umberto I – Lancisi – Salesi” of Ancona, Ancona, Italy

**Keywords:** Aortic surgery, Ischemia/reperfusion injury, Anaesthesia, Haemodynamics, Microcirculation

## Abstract

**Background:**

In vascular surgery with aortic cross-clamping, ischemia/reperfusion injury induces systemic haemodynamic and microcirculatory disturbances. Different anaesthetic regimens may have a varying impact on tissue perfusion. The aim of this study was to explore changes in microvascular perfusion in patients undergoing elective open abdominal aortic aneurysm repair under balanced or total intravenous anaesthesia.

**Methods:**

Prospective observational study. Patients undergoing elective open infrarenal abdominal aortic aneurysm repair received balanced (desflurane + remifentanil, *n* = 20) or total intravenous anaesthesia (TIVA, propofol + remifentanil using target-controlled infusion, n = 20) according to the clinician’s decision. A goal-directed haemodynamic management was applied in all patients. Measurements were obtained before anaesthesia induction (baseline) and at end-surgery and included haemodynamics, arterial/venous blood gases, sublingual microvascular flow and density (incident dark field illumination imaging), peripheral muscle tissue oxygenation and microcirculatory reactivity (thenar near infrared spectroscopy with a vascular occlusion test).

**Results:**

The two groups did not differ for baseline characteristics, mean aortic-clamping time and requirement of vasoactive agents during surgery. Changes in mean arterial pressure, systemic vascular resistance index, haemoglobin and blood lactate levels were similar between the two groups, while the cardiac index increased at end-surgery in patients undergoing balanced anaesthesia. The sublingual microcirculation was globally unaltered in the TIVA group at end-surgery, while patients undergoing balanced anaesthesia showed an increase in the total and perfused small vessel densities (from 16.6 ± 4.2 to 19.1 ± 5.4 mm/mm^2^, *p* < 0.05). Changes in microvascular density were negatively correlated with changes in the systemic vascular resistance index. The area of reactive hyperaemia during the VOT increased in the balanced anaesthesia group (from 14.8 ± 8.1 to 25.6 ± 14.8%*min, *p* < 0.05). At end-surgery, the tissue haemoglobin index in the TIVA group was lower than that in the balanced anaesthesia group.

**Conclusions:**

In patients undergoing elective open abdominal aortic aneurysm repair with a goal-directed hemodynamic management, indices of sublingual or peripheral microvascular perfusion/oxygenation were globally preserved with both balanced anaesthesia and TIVA. Patients undergoing balanced anaesthesia showed microvascular recruitment at end-surgery.

**Trial registration:**

NCT03510793, https://www.clinicaltrials.gov, date of registration April 27th 2018, retrospectively registered.

## Background

Ischemia/reperfusion (I/R) injury is common in patients undergoing aortic clamping for vascular surgery and leads to systemic inflammation and organ dysfunction [1–3]. The production of pro-inflammatory molecules and oxidative stress increase especially in the reperfusion phase and are responsible for microvascular alterations similar to those observed during sepsis [4–6]. These include an impairment in blood flow, capillary shunting with reduction in microcirculatory density and increased perfusion heterogeneity, resulting in a mismatch between oxygen delivery and consumption [4, 5]. The severity of microcirculatory dysfunction increases along with the duration of the ischemic phase [2, 3, 7].

A number of studies explored how drugs can modulate microvascular perfusion in different disease states. In septic patients with impaired blood flow auto-regulation, vasoactive agents may have limited potential for microvascular recruitment [8]: the use of norepinephrine titrated to reach a mean arterial pressure (MAP) > 65 mmHg was unable to improve microcirculatory perfusion during septic shock [9, 10]. Dobutamine increased intestinal microvascular blood flow in animal models of sepsis [11] but failed to restore capillary perfusion in patients with septic shock [12]. Conversely, after cardiac surgery a rise in MAP by norepinephrine infusion induced an increase in splanchnic oxygen extraction without altering the intestinal mucosal perfusion, possibly because of autoregulation phenomenon [13–15].

Anaesthetics can affect microvascular perfusion as well. Propofol causes vasodilation by stimulating nitric oxide production: this may result in microvascular shunting with reduction in capillary density, increased blood flow heterogeneity and reduced tissue oxygen delivery [16–18]. Volatile anaesthetics also cause dose-dependent vasodilation [19, 20]. In anesthetised dogs, desflurane, unlike isoflurane and halothane, was able to maintain myocardial, hepatic, intestinal and skeletal muscle blood flow [21]. In patients undergoing thoracoscopic surgery, desflurane-remifentanil anaesthesia was associated with better microvascular perfusion as compared to propofol-remifentanil anaesthesia [22]. In female patients undergoing breast surgery, the use of sevoflurane, unlike propofol, was associated with a significant decrease in the capillary filtration coefficient, suggesting a lower risk of interstitial oedema and tissue perfusion alteration [23]. Microcirculatory dysfunction was found in different surgical settings despite the optimization of systemic hemodynamic parameters [24] and was associated with the development of post-operative complications [25].

We aimed to verify whether desflurane might have a better impact on the microcirculation as compared to propofol in patients at risk for I/R injury due to aortic surgery. The goal of this study was to explore changes in sublingual and peripheral muscle microcirculation in patients undergoing elective open abdominal aortic aneurysm repair under balanced or total intravenous anaesthesia (TIVA).

## Methods

The study was approved by our local ethic committee of Azienda Ospedaliera Universitaria “Ospedali Riuniti” of Ancona in Italy (ClinicalTrials.gov identifier: NCT03510793, www.clinicaltrials.gov, principal investigator: Prof. Abele Donati, date of registration: April 27th 2018, retrospectively registered). A written informed consent was obtained from all patients before enrolment. This was designed as a prospective observational study on 40 patients undergoing elective open infrarenal abdominal aortic aneurysm repair with prosthetic aorto-aortic or aorto-bisiliac bypass under general anaesthesia. The study was performed at Vascular Surgery of the Azienda Ospedaliera Universitaria “Ospedali Riuniti” of Ancona in Italy between September 2013 and November 2017.

Inclusion criteria were: elective infrarenal abdominal aortic open repair; use of a protocol of intraoperative hemodynamic goal-directed therapy; American Society of Anaesthesiology (ASA) class ≤ III. Exclusion criteria were: age < 18 years, pregnancy, endovascular aneurysm repair, presence of infections, trauma, emergency surgery.

Patients received balanced anaesthesia or TIVA based on the attending physician’s decision, resulting in two study groups of 20 patients each. The choice of the anaesthetic regimen was based exclusively on the physician preference and was taken before and independently of the patient’s enrolment, the anaesthesiologist was unaware of baseline microcirculatory measurements. The patients’ distribution between the two groups was initially due to chance; the enrolment was then stopped in the balanced anaesthesia group once the required sample size (*n* = 20) had been reached, in order to achieve the same number of patients in the TIVA group. Patients undergoing balanced anaesthesia received desflurane (with an inspiratory oxygen fraction of 0.4–0.5) and remifentanil, administered as a target-controlled infusion (Minto model). TIVA was performed with a target-controlled infusion of propofol (Schnider model) and remifentanil (Minto model). In all patients, anaesthetic depth was monitored with spectral entropy and the anaesthetic dose was titrated in order to maintain a state entropy value of 35 to 45%, resulting in an age-adjusted minimum alveolar concentration of 0.8 for desflurane and an estimated effect-site target concentration (Ce) of 2–4 mcg/ml and 2.5–5 ng/ml for propofol and remifentanil, respectively. Neuromuscular blockade was achieved with a bolus of 0.6 mg/kg rocuronium at anaesthesia induction; additional boluses (10 mg) were provided based on train-of-four neuromuscular monitoring. Haemodynamic parameters were monitored in all patients with Flotrac/Vigileo (Edwards Lifesciences); arterial and central venous blood gases were monitored according to routine clinical practice. A protocol of intraoperative goal-directed therapy was applied in all patients for haemodynamic optimization, according to standard care in our unit [26]. This includes: first, stroke volume optimization by fluid therapy (targeting an increase in stroke volume < 10% after a 200 ml fluid bolus over 5 min); second maintenance of a MAP > 70 mmHg by titrated vasopressor administration; third, maintenance of a cardiac index (CI) ≥2.5 ml/kg per m^2^ by titrated inotropic therapy [26].

Measurements were collected at baseline (before induction of anaesthesia) and at end-surgery (before anaesthesia discontinuation) and included haemodynamic parameters, arterial and central venous blood gases and assessment of the sublingual microcirculation and peripheral muscle tissue oxygenation and microvascular reactivity.

### Evaluation of the sublingual microcirculation

The sublingual microcirculation was assessed with incident dark field (IDF) imaging that is incorporated into a handheld video microscope (CytoCam, Braedius, Amsterdam, The Netherlands) and enables a real time visualisation of microvascular blood flow. Details on this technique have been described elsewhere [27]. After gentle removal of saliva and other secretions with a gauze, the probe was applied on the sublingual mucosa. Three videos of 10 s’ duration were recorded on three different areas with adequate focus and contrast. All efforts were made to avoid pressure artifacts. Videos were analysed offline with a dedicated software (Automated Vascular Analysis v3.0, Microvision Medical, Amsterdam, The Netherlands). For small (diameter < 20 μm) and medium (diameter 20–50 μm) vessels, we calculated the Total Vessel Density (TVD), Perfused Vessel Density (PVD), De Backer score, Percentage of Perfused Vessels (PPV), Microvascular Flow Index (MFI) and Flow Heterogeneity Index (FHI), as previously described [28].

### Evaluation of tissue oxygenation and microvascular reactivity

Near-infrared spectroscopy (NIRS) (InSpectra™ Model 650, Hutchinson Technology Inc., Hutchinson, MN, USA) was applied on the thenar eminence with a 15-mm sized probe to measure peripheral muscle tissue oxygen saturation (StO_2_) and tissue haemoglobin index (THI) [29] before and during a vascular occlusion test (VOT), as described previously [30]. This was performed by inflating a sphygmomanometer cuff placed on the forearm to 50 mmHg above the systolic blood pressure. Arterial inflow was arrested until the StO_2_ decreased to 40%. StO_2_ was recorded during the ischemic and the reperfusion phases until stabilization [31]. NIRS-derived parameters were calculated by using a software package (version 3.02 InSpectra Analysis Program; Hutchinson Technology Inc.). The StO_2_ downslope was calculated from the regression line of the StO_2_ decay after occlusion, providing an index of tissue oxygen extraction rate [31]. The desaturation slope may vary during the ischaemic phase of the VOT, becoming more or less steep before reaching the 40% StO_2_ threshold: the inflection point was identified and the downslope was calculated separately for the first and the last part of the desaturation curve (downslope 1 and downslope 2, respectively), as previously described [32]. Whenever a change in the slope was not observed, the desaturation curve was divided into two halves for the calculation of the two downslope values. The delta-downslope was calculated as the difference between the last and the first part of the desaturation slope (downslope 2 - downslope1), so that a positive value indicated a flattening in the second part of the slope (slower StO_2_ decay) [32]. The StO_2_ upslope during the reperfusion phase and the area under the curve of the StO_2_ (AUC StO_2_) during the hyperaemic response were calculated as indices of microcirculatory reactivity [31].

### Statistical analysis

The analysis was performed using Graphpad Prism 6 (GraphPad Software, La Jolla, CA, USA). The Kolmogorov-Smirnov test was used to check the normality of distribution. Data were expressed as mean ± standard deviation or median [1st-3rd quartile], as appropriate. A chi-square test or Student’s t test (or Mann Whitney U test) were applied for between-group comparisons of nominal or continuous variables, as appropriate. A two-way analysis of variance (ANOVA) with the Sidack’s multiple comparison test was used whenever possible. Alternatively, the Wilcoxon and Mann-Whitney U test were used for non-normally distributed variables for comparisons between the two time points in the same group and between the two groups at the same time point. The Spearman rho was calculated to evaluate the correlation between variables. A (two-sided) *p* value < 0.05 was used to indicate statistical significance. For sample size calculation, we considered a difference in MFI at end-surgery of 0.4 (standard deviation 0.4) as clinically relevant [28]. A sample size of 16 patients per group was sufficient to detect such a difference with a power of 80% and an alpha error of 0.05. We included 40 patients (20 per group) to allow for missing data or dropouts.

## Results

**Fig. 1:**
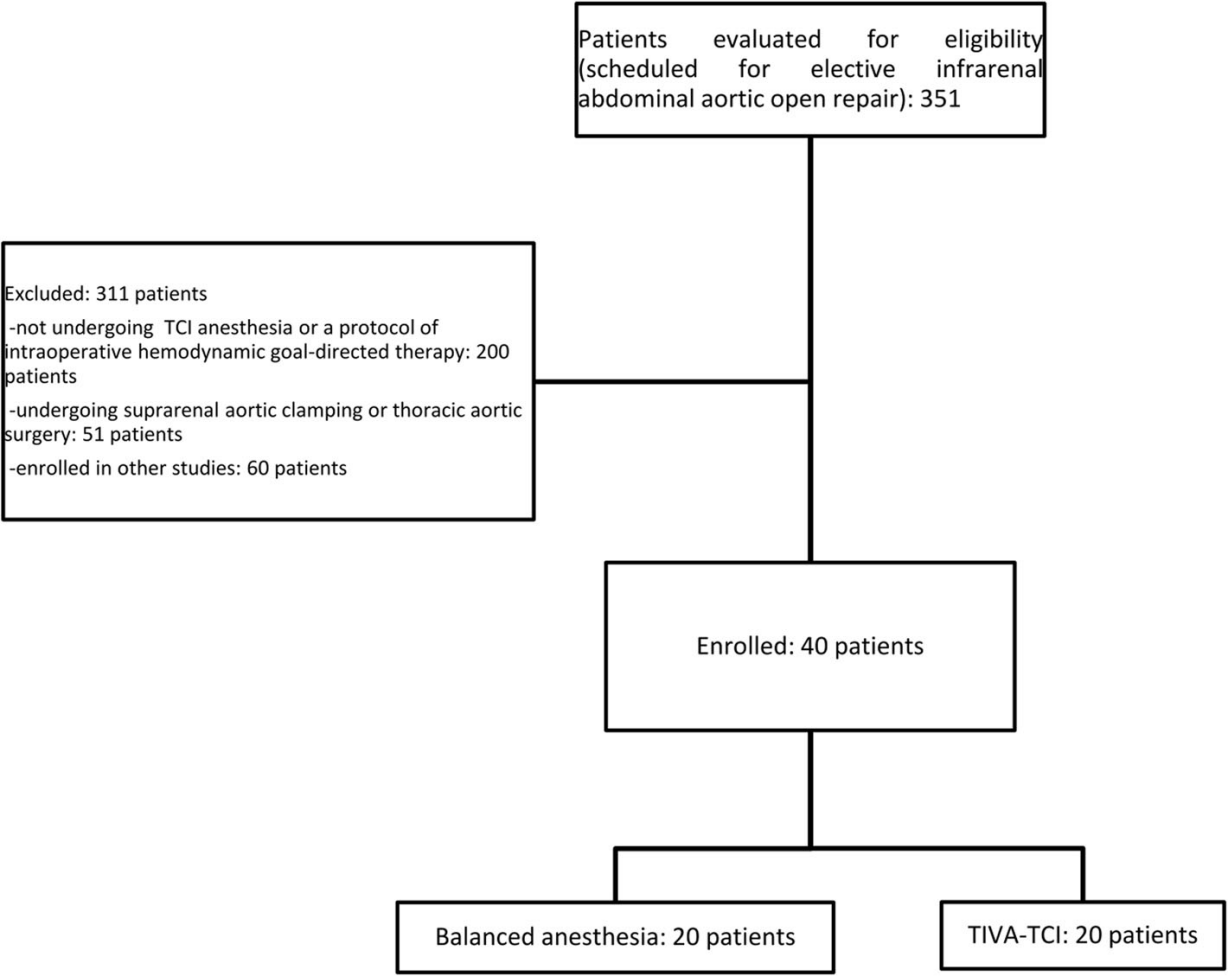
Study flow diagram

Figure 1 shows the study flow diagram, with details on the number of patients evaluated for eligibility and reasons for exclusion. The two groups did not differ for age, gender distribution, comorbidities, ASA score, and intraoperative data including clamping time, fluid balance at end-surgery and vasoactive agent requirements (Table 1). Changes in haemodynamics and blood gases from baseline to end-surgery are reported in Table 2. MAP and systemic vascular resistance index (SVRI) decreased in both groups. The CI increased in patients undergoing balanced anaesthesia, while it remained substantially stable in the TIVA group. The two groups showed similar changes in haemoglobin (Hb), central venous oxygen saturation (ScvO_2_), arterial lactate levels and arterial base excess.

**Table 1:** General and intraoperative data for the two groups of patients receiving balanced anaesthesia or total intravenous anaesthesia

	Balanced anaesthesia	TIVA	*p*
Age (years)	75 ± 7	74 ± 6	0.464
Gender (nr, % of males)	16, 80%	18, 90%	0.376
ASA score (nr, %)			0.127
*2*	13, 65%	18, 90%	
*3*	7, 35%	2, 10%	
Comorbidities (nr, %)			
*Arterial hypertension*	20, 100%	18, 90%	0.149
*Cardiopathy*	6, 30%	10, 50%	0.197
*Renal Failure*	3, 15%	5, 25%	0.429
*COPD*	12, 60%	8, 40%	0.206
*Dyslipidaemia*	10, 50%	10, 50%	0.999
*Vasculopathy (lower limbs or carotid)*	10, 50%	8, 40%	0.525
Clamping time (min)	65 ± 29	69 ± 31	0.691
Fluid balance (ml)	−200 [− 1100 − + 150]	− 195 [−670 − + 300]	0.657
Norepinephrine			
*number, %*	20, 100%	20, 100%	0.999
*mean dosage (mcg/kg/min)*	0.15 [0.08–0.20]	0.12 [0.09–0.15]	0.466
Dobutamine			
*number, %*	13, 65%	16, 80%	0.288
*mean dosage (mcg/kg/min)*	2.00 [1.23–3.00]	2.39 [1.67–3.92]	0.416
Esmolol (nr, %)	14, 70%	10, 50%	0.197
Levosimendan (nr, %)	3, 15%	5, 25%	0.429
Blood transfusions			
*number, %*	10, 50%	10, 50%	0.999
*average amount (ml)*	540 [450–1060]	580 [450–780]	0.837

Data are expressed as mean ± standard deviation or median [1st-3rd quartile], as appropriate

Cardiopathy was defined by the presence of echocardiographic alterations including left ventricular hypertrophy and valvulopathy, or a history of coronary artery disease and/or heart failure and reduced left ventricular ejection fraction. Renal failure was defined by the presence of an estimated glomerular filtration rate lower than 60 ml/min/1.73mq

*TIVA* total intravenous anaesthesia, *ASA* American Society of Anesthesiology, *COPD* chronic obstructive pulmonary disease

**Table 2:** Changes in haemodynamics and blood gases from baseline to end-surgery in the two groups of patients receiving balanced anaesthesia or total intravenous anaesthesia

	Balanced anaesthesia		TIVA		p for interaction (2-way ANOVA)
	baseline	end-surgery	baseline	end-surgery	
MAP (mmHg)	94 ± 18	86 ± 13*	92 ± 14	81 ± 12**	0.642
HR (bpm)	67 ± 13	71 ± 11	70 ± 13	71 ± 14	0.521
CI (ml/m^2^/min)	2.4 ± 0.7	3.2 ± 0.6***	2.6 ± 0.5	2.8 ± 0.7	0.005
SVRI (dyn*s*cm^− 5^)	2800 [2352–3224]	1839 [1691–2085]***	2488 [2203–2952]	2119 [1754–2350]***	–
pH	7.42 [7.39–7.47]	7.40 [7.38–7.43]	7.43 [7.42–7.44]	7.41 [7.37–7.45]	–
PaO_2_ (mmHg)	70 [62–88]	159 [145–207]**	72 [63–166]	194 [134–235]***	–
PaCO_2_ (mmHg)	38 ± 4	38 ± 4	37 ± 3	39 ± 5*	0.027
Base excess (mEq/l)	0.5 [−0.3, + 2.5]	− 0.4 [− 3.2, + 1.2]**	0.5 [− 1.4, + 1.2]	− 0.4 [− 1.9, + 2.4]	–
ScvO_2_ (%)	72 [69–78]	90 [82–92]***	79 [70–90]#	91 [86–94]**	–
Lactate (mmol/l)	0.6 [0.6–0.8]	1.3 [1.1–1.9]***	0.7 [0.6–1.0]	1.3 [1.0–1.5]***	–
Hb (g/dl)	13.3 ± 2.0	11.8 ± 0.8***	13.4 ± 1.6	11.8 ± 1.3***	0.793
Glucose (mg/dl)	96 [86–107]	121 [105–131]***	102 [95–112]	125 [106–141]***	–

*p < 0.05 **p < 0.01 ***p < 0.001 versus baseline, #*p* < 0.05 versus time-matched balanced anaesthesia. Two-way Analysis of Variance (ANOVA) with Sidack’s multiple comparisons test or Wilcoxon and Mann-Whitney U test, as appropriate. Data are expressed as mean ± standard deviation or median [1st-3rd quartile], as appropriate. *TIVA* total intravenous anaesthesia, *MAP* mean arterial pressure, *HR* heart rate, *CI* cardiac index, *SVRI* systemic vascular resistance index, *PaO*_*2*_ arterial oxygen tension, *PaCO*_*2*_ arterial carbon dioxide tension, *ScvO*_*2*_ central venous oxygen saturation, *Hb* haemoglobin

**Fig. 2:**
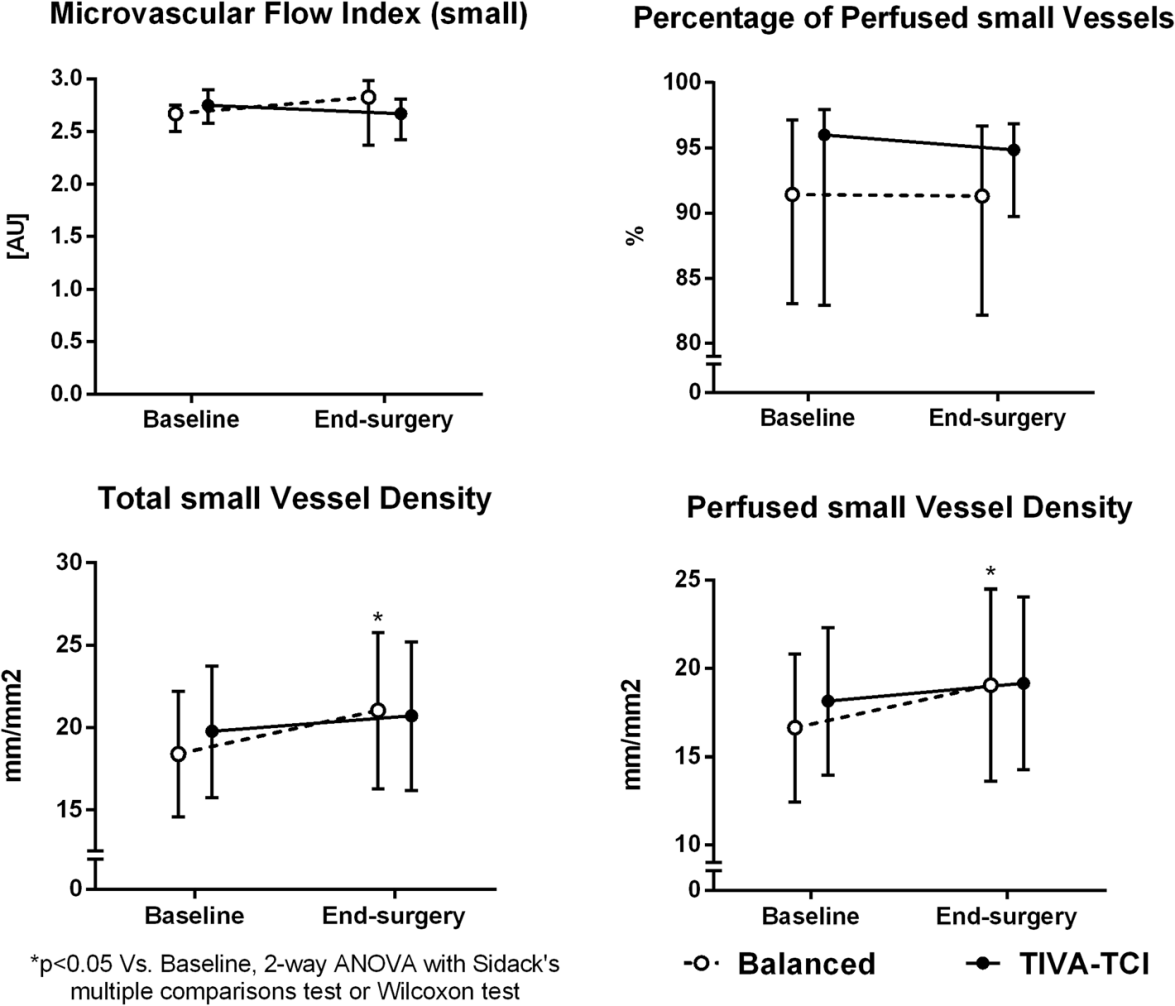
Changes in sublingual microvascular parameters from baseline to end-surgery in the two groups of patients receiving balanced anaesthesia or total intravenous anaesthesia (TIVA)

Changes in microcirculatory and NIRS-derived parameters are shown in Table 3. The TVD and PVD for small vessels increased at end-surgery in the balanced anaesthesia group, while the small vessel MFI and PPV remained unaltered in both groups (Fig. 2). At both time points, the TVD and PVD for medium vessels were lower in the TIVA group as compared to those in the balanced anaesthesia group (Table 3). Changes in microvascular density were negatively correlated with changes in SVRI (TVD for small vessels: *r* = − 0.479 *p* = 0.002; PVD for small vessels: *r* = − 0.451 *p* = 0.003). These correlations remained if considering the two groups separately, although appearing weaker in the TIVA group (TVD for small vessels: *r* = − 0.453 *p* = 0.045, PVD for small vessels: *r* = − 0.362 *p* = 0.116) as compared to those observed the balanced anaesthesia group (TVD for small vessels: *r* = − 0.526 *p* = 0.017, PVD for small vessels: *r* = − 0.515 *p* = 0.020). No correlation was found between microvascular parameters and CI, MAP or mean norepinephrine dose during surgery. In all patients, a negative correlation was found between changes in SVRI and mean norepinephrine dose (*r* = − 0.387, *p* = 0.022).

**Table 3:** Changes in sublingual microcirculation and NIRS-derived parameters from baseline to end-surgery in the two groups of patients receiving balanced anaesthesia or total intravenous anaesthesia

	Balanced anaesthesia		TIVA		p for interaction (2-way ANOVA)
	Baseline	end-surgery	baseline	end-surgery	
MFI (small vessels) [AU]	2.67 [2.50–2.75]	2.83 [2.37–2.98]	2.75 [2.58–2.90]	2.67 [2.42–2.81]	–
Abnormal MFI^a^ (n, %)	9, 45%	6, 30%	7, 35%	9, 45%	–
FHI [AU]	0.15 [0.00–0.29]	0.18 [0.02–0.36]	0.23 [0.09–0.28]	0.19 [0.10–0.38]	–
TVD (small) (mm/mm2)	18.4 ± 3.8	21.0 ± 4.7*	19.8 ± 4.0	20.7 ± 4.5	0.216
TVD (medium) (mm/mm2)	1.5 [0.9–2.2]	1.2 [1.1–1.5]	0.8 [0.6–1.1]##	0.5 [0.1–0.8]###	–
PVD (small) (mm/mm2)	16.6 ± 4.2	19.1 ± 5.4*	18.1 ± 4.2	19.2 ± 4.9	0.302
PVD (medium) (mm/mm2)	1.5 [0.9–2.2]	1.2 [1.1–1.5]	0.8 [0.6–1.1]##	0.5 [0.1–0.8]###	–
PPV (small, %)	91 [83–97]	91 [82–97]	96 [83–98]	95 [90–97]	–
StO_2_ (%)	83 ± 5	86 ± 7	82 ± 6	85 ± 9	0.975
StO_2_ Downslope 1 (%/min)	−8.0 [−10.8, −6.8]	−7.8 [− 10.4, − 6.9]	− 8.8 [− 12.0, − 7.8]	−8.1 [− 11.1, − 5.9]**	–
StO_2_ Downslope 2 (%/min)	−8.2 ± 3.3	− 8.0 ± 4.8	−8.2 ± 2.8	− 8.0 ± 3.4	0.962
Delta Downslope (2–1) (%/min)	1.1 [− 0.5, 2.4]	−0.1 [− 1.6, 1.8]	0.9 [− 0.8, 3.9]	−0.7 [− 1.4, 1.0]	–
StO_2_ Upslope (%/min)	226 ± 64	206 ± 103	264 ± 84	247 ± 77	0.900
AUC StO_2_ (%*min)	14.8 ± 8.1	25.6 ± 14.8*	20.4 ± 6.0	26.1 ± 19.1	0.359
THI [AU]	15.2 ± 2.0	15.3 ± 3.1	14.6 ± 3.3	12.8 ± 3.6#	0.140

*p < 0.05 **p < 0.01 versus baseline, #*p* < 0.05 ##*p* < 0.01 ###*p* < 0.001 versus time-matched balanced anesthesia. Two-way Analysis of Variance (ANOVA) with Sidack’s multiple comparisons test or Wilcoxon and Mann-Whitney U test, as appropriate. Data are expressed as mean ± standard deviation or median [1st-3rd quartile], as appropriate

^a^An abnormal MFI was defined as an MFI lower than 2.6 [25]

*NIRS* near infrared spectroscopy, *TIVA* total intravenous anaesthesia, *MFI* microvascular flow index, *AU* arbitrary unit, *FHI* flow heterogeneity index, *TVD* total vessel density, *PVD* perfused vessel density, *PPV* percentage of perfused vessels, *StO*_*2*_ tissue oxygen saturation, *AUC StO*_*2*_ area under the curve of StO_2_, *THI* tissue haemoglobin index

**Fig. 3:**
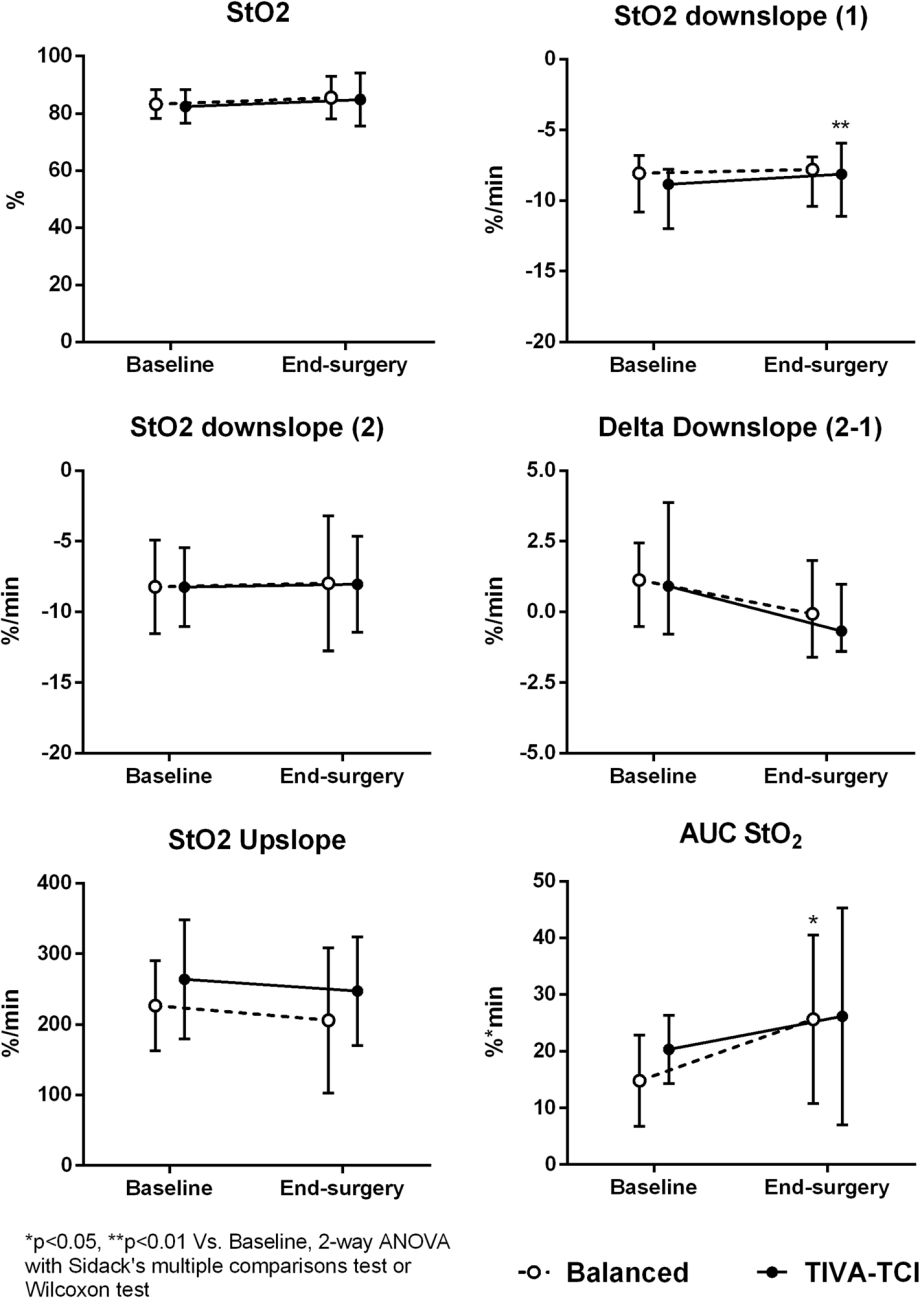
Changes in NIRS-derived parameters from baseline to end-surgery in the two groups of patients receiving balanced anaesthesia or total intravenous anaesthesia (TIVA). *StO*_*2*_ tissue oxygen saturation, *AUC StO*_*2*_ area under the curve of StO_2_ (area of hyperaemia)

**Fig. 4:**
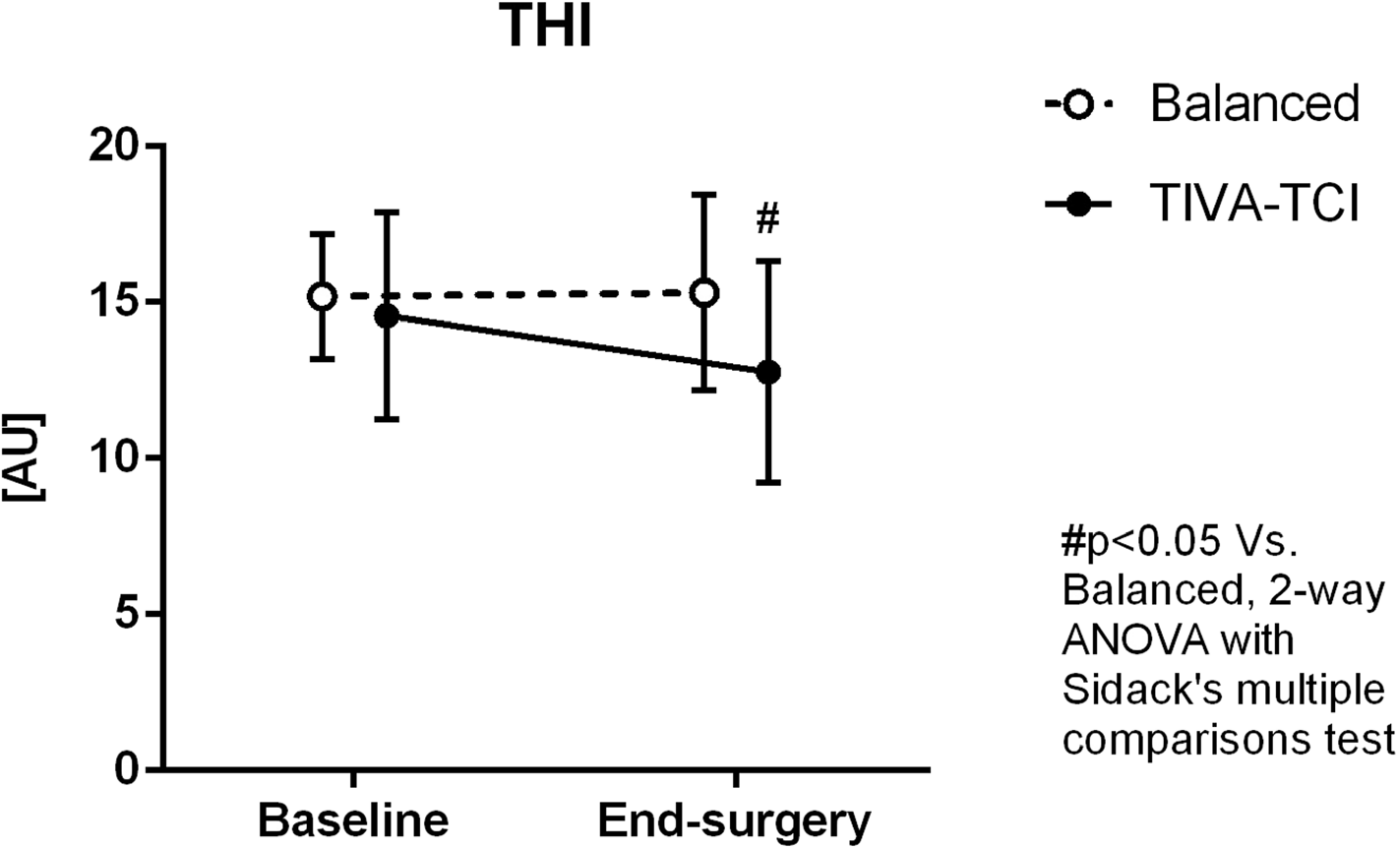
Changes in the tissue haemoglobin index (THI) from baseline to end-surgery in the two groups of patients receiving balanced anaesthesia or total intravenous anaesthesia (TIVA)

The StO_2_ did not vary from baseline to end-surgery in either group (Fig. 3). The StO_2_ downslope 1 increased in the TIVA group at end-surgery (indicating a slower desaturation rate in the initial part of the ischemic phase), while it remained stable in the balanced anaesthesia group (Fig. 3). The StO_2_ downslope 2, delta-downslope and StO_2_ upslope did not vary in either group (Table 3). The AUC StO_2_ increased at end-surgery in the balanced anaesthesia group (Fig. 3). The THI tended to decrease and was significantly lower at end-surgery in the TIVA group, while it remained stable in the balanced anaesthesia group (Fig. 4).

## Discussion

In this prospective observational study on 40 patients undergoing elective open abdominal aortic aneurysm repair with an intraoperative goal-directed haemodynamic optimization, we aimed to explore whether different anaesthetic regimens could have a varying impact on microvascular perfusion in a condition of potential I/R injury. Our first finding is that both balanced anaesthesia and TIVA were associated with overall maintenance of microvascular perfusion and tissue oxygenation. Patients receiving balanced anaesthesia showed an increase in microvascular density, which was inversely related to a reduction in systemic vascular resistance. In patients undergoing TIVA we observed a minor reduction in the skeletal muscle tissue oxygen extraction rate during a VOT and lower THI at end-surgery as compared to that observed in the balanced anaesthesia group. Balanced anaesthesia was associated with an increase in microvascular reactivity, as reflected by a greater area of hyperaemia after the VOT as compared to that seen at baseline.

The activation of the inflammatory response may occur early during aortic surgery [33] with maximal production of oxygen free radicals within 5 min of lower-limb reperfusion [34]. Previous studies showed that oxidative stress during supraceliac or infrarenal aortic cross clamping and reperfusion leads to distributive alterations in microvascular oxygenation and perfusion of splanchnic organs [35]. Heterogeneous flow distribution is characterized by capillaries with stagnant blood cells next to capillaries with normal flow [36] and can be found in microvascular beds far from the site of direct ischemic damage [37]. In rats subjected to I/R injury of the small bowel, the intestinal microvascular blood flow was reduced early in the first 10 min of reperfusion and failed to normalize in the first 2 h [38]. Microcirculatory disturbances during I/R injury may occur independently of changes in macro-haemodynamics and tissue hypo-perfusion may persist despite a normalisation of systemic cardiocirculatory parameters due to a loss of haemodynamic coherence [39]. In our study, we did not find an impairment in microvascular perfusion at end-surgery. We hypothesize that intraoperative hemodynamic optimization with the routine use of a protocol of goal-directed therapy prevented the development of microvascular derangements. In pigs subjected to colon anastomosis, a goal-directed colloid administration was able to improve microvascular perfusion and oxygenation of healthy and perianastomotic colon [40]. Although we could not find any correlation with MAP or CI, changes in sublingual capillary density were inversely related with changes in systemic vascular resistance, reflecting a certain degree of coherence between the macro- and micro-haemodynamic responses in our population [41].

Sublingual microvascular density increased at end-surgery in patients receiving balanced anaesthesia, while it remained unaltered in the TIVA group. It cannot be excluded that the microvascular changes observed were the effect of vasodilating metabolites (e.g. adenosine monophosphate, hypoxanthine) released during reperfusion, however we would expect this reactive hyperaemia to disappear in a late reperfusion phase at end-surgery [3]. Even if the observational design of our study prevents to define cause-effect relationships, the between-group homogeneity in baseline and intraoperative characteristics (including clamping time, fluid balance, blood losses, type and dose of vasoactive agents) would corroborate the hypothesis of direct and separate actions of desflurane and propofol on the microcirculation.

Previous studies showed that both propofol and sevoflurane can exert a protective effect towards I/R injury by modulating the inflammatory response, reducing oxidative stress and tissue apoptosis [42]. Halogenated anaesthetics induce peripheral vasodilation [43] and reduce vascular permeability [23], thus favouring microvascular recruitment and tissue oxygen diffusion. On the other hand, propofol can modulate the inflammatory response and oxidative stress [44], thus increasing tolerance to hypoxia also in tissues far from those directly exposed to ischemia [45]. In this study, desflurane may have been responsible for vasodilation and vascular recruitment in the balanced anaesthesia group due to dilation of resistive arterioles. The increased area of hyperaemia after the VOT would also be consistent with a greater microvascular recruitment following the ischemic challenge in the balanced anaesthesia group.

The rationale of evaluating the microcirculatory response to therapies is that improving microvascular perfusion will guarantee tissue oxygenation and prevent the development of organ dysfunction. In a previous study in patients undergoing abdominal aortic aneurysm surgery, we showed that portal lactate, intramucosal sigmoid pH and arterial-sigmoid delta pCO_2_ (as indices of splanchnic hypo-perfusion) were able to predict the occurrence of post-operative complications [46]. In the present study, we could not show a better impact on anaerobic metabolism of desflurane or propofol, as a clinically relevant hyper-lactataemia was not found in either group at end-surgery. This was consistent with the absence of major microcirculatory alterations in both the balanced anaesthesia and the TIVA groups.

While the StO_2_ remained stable in both groups, the desaturation rate in the first part of the ischemic challenge decreased at end-surgery in patients undergoing TIVA. This may result from a reduction in peripheral microvascular density and would be consistent with the lower THI at end-surgery as compared to that seen in the balanced anaesthesia group, which seems to be related to a lower tissue perfusion rather than to a reduction in Hb levels as these remained similar in the two groups. Baseline differences in StO_2_ downslope between the groups, although not significant, could also explain our results: the observed variation may only depend upon a rebalance in the metabolic pattern in the TIVA group without a dysfunctional meaning. In critically ill patients, we showed that a decrease in the desaturation rate in the last part of the ischaemic phase of the VOT is associated with worse outcome and could suggest impaired microcirculatory auto-regulation [32]. In this study, the StO_2_ downslope was again similar between the groups in the final part of vascular occlusion and the delta-downslope did not differ between the two groups. Therefore, the type of anaesthesia may not significantly affect peripheral microcirculatory auto-regulation.

### Limitations of the study

First, the observational design does not allow drawing any cause-effect relationship between the type of anaesthesia and the microvascular changes observed. Moreover, lack of randomization and the relatively small sample size prevented to control for possible confounding factors. This study was designed as a preliminary exploratory analysis with the aim to detect any possible influence of different anaesthetic regimens on microvascular perfusion alterations during aortic surgery. Future randomized trials are needed to confirm our findings. Second, we cannot exclude that baseline differences in some parameters influenced our results. Even if the main haemodynamic parameters were similar between the groups at baseline, the lower ScvO_2_ in the balanced anaesthesia group, as well as differences in baseline microvascular medium vessel density, may indicate a higher initial tissue O_2_ demand/consumption. However, baseline microvascular small vessel density and NIRS-derived parameters were similar between the two groups, suggesting a similar pattern of tissue perfusion. Third, a relevant number of patients among those assessed for eligibility was excluded, therefore we cannot exclude the presence of a selection bias. Lastly, the microcirculation was globally preserved in both groups, suggesting an overall low prevalence of I/R injury syndrome in our sample. Our study may have been underpowered to detect significant microvascular derangements following aortic surgery. Moreover, monitoring of the sublingual microcirculation may not be sensitive enough to detect tissue perfusion alterations in splanchnic organs [47]. Unfortunately, we did not evaluate the incidence of organ dysfunction or post-operative complications.

## Conclusions

In patients undergoing open abdominal aortic aneurysm repair under general anaesthesia with a protocol of intraoperative hemodynamic goal-directed therapy, microvascular perfusion and peripheral tissue oxygenation were generally preserved, however the use of balanced anaesthesia was associated with increased microvascular density and reactivity, while these remained unaltered with TIVA. Further studies are needed to clarify whether the choice of different anaesthetic regimens may contribute to prevent I/R injury in aortic surgery.

## Abbreviations

*ASA*: American Society of Anaesthesiology
*AUC StO*_*2*_: Area under the hyperaemic phase
*CI*: Cardiac index
*Cp*: Plasma target concentration
*FHI*: Flow heterogeneity index
*Hb*: Haemoglobin
*I/R*: Ischemia/reperfusion
*IDF*: Incident Dark Field
*MAP*: Mean arterial pressure
*MFI*: Microvascular flow index
*NIRS*: Near infrared spectroscopy
*PPV*: Percentage of perfused vessels
*PVD*: Perfused vessel density
*ScvO*_*2*_: Central venous oxygen saturation
*StO*_*2*_: Tissue oxygen saturation
*SVRI*: Systemic vascular resistance index
*THI*: Tissue haemoglobin index
*TIVA*: Total intravenous anaesthesia
*TVD*: Total vessel density
*VOT*: Vascular occlusion test
